# Resistant starch selectively depletes a putative pathobiont-enriched gut microbial module: evidence from multiple dietary fiber intervention cohorts

**DOI:** 10.3389/fnut.2026.1845191

**Published:** 2026-05-20

**Authors:** Biao Dong, Mai Ye, Zhenjiang Zech Xu, Xiaochang Huang, Yichen Hu

**Affiliations:** 1 State Key Laboratory of Food Science and Technology, Nanchang University, Nanchang, China; 2 School of Mathematics and Computer Sciences, Nanchang University, Nanchang, China

**Keywords:** gut immune microenvironment, gut microbiota, inflammatory bowel disease, multi-cohort study, precision nutrition, resistant starch

## Abstract

**Objectives:**

Dietary fiber, particularly resistant starch (RS), has been proposed to modulate the gut microbiota and immune microenvironment; however, individual intervention studies yield inconsistent results due to inter-study heterogeneity. This study aimed to identify reproducible gut microbiota responses to RS across multiple independent cohorts and explore the ecological basis by which RS shapes microbial community structure.

**Methods:**

Gut microbiota composition was analyzed in 586 paired fecal samples collected from healthy adults in multiple independent cohorts undergoing RS dietary interventions. All datasets were processed through a unified bioinformatics pipeline to minimize technical variability. Differential abundance analysis, co-occurrence network analysis, and validation against inflammatory bowel disease (IBD) populations were performed to identify reproducible microbial responses and assess their potential clinical relevance.

**Results:**

RS supplementation was associated a consistent reduction in alpha diversity and a reproducible, unidirectional taxonomic response: 22 specific taxa were consistently depleted across cohorts, including putative immunostimulatory pathobionts such as *Ruminococcus gnavus*, without universally enriching any single beneficial species. PICRUSt2-based functional predictions showed limited KO-level changes, whereas CAZyme profiles showed a non-significant trend toward increased carbohydrate-degradation potential, with no individual CAZyme family surviving FDR correction.. Machine learning classification achieved moderate cross-cohort discrimination (leave-one-study-out AUROC ≈ 0.68). Cross-sectional validation in eight inflammatory bowel disease cohorts showed that RS-depleted taxa were significantly enriched in Crohn’s disease and ulcerative colitis (*p* < 0.01).

**Conclusion:**

Across cohorts, RS intake was consistently associated with a selective ecological filter that consistently depletes a set of microbial taxa including putative immunostimulatory pathobionts, providing a mechanistic basis for its proposed role in dietary modulation of the gut immune microenvironment. These consistently depleted taxa may serve as candidate biomarkers to identify individuals most likely to benefit from RS-based dietary fiber interventions, while their utility for predicting individual responsiveness before intervention remains to be tested.

## Introduction

Dietary fibers, which are defined as carbohydrates that resist digestion by the small intestine, are crucial for maintaining human health. This category includes compounds such as resistant starch (RS) (1, 2). High-fiber diets are associated with a reduced risk or amelioration of numerous illnesses, including constipation, obesity, diabetes, high cholesterol, and heart disease (3–5). Furthermore, the benefits of dietary fiber are linked to improved mineral absorption, better insulin responses, reduced intestinal permeability, and a more robust immune system defense (6, 7). Many of these benefits are driven by microbial fermentation of dietary fiber, which generates metabolites such as SCFAs that regulate immune function, gut barrier integrity, and glucose metabolism (8). This, in turn, alters broader microbial activity through ecological processes such as metabolic coupling and competition.

Notably, resistant starch (RS) can be metabolized by the gut microbiota to produce short-chain fatty acids (SCFAs). These metabolites are key immune signaling mediators. By engaging GPR41/GPR43 pathways and suppressing histone deacetylase (HDAC) activity, SCFAs can regulate the intestinal immune microenvironment, contributing to the maintenance of mucosal barrier integrity and the induction of regulatory T cell (Treg) differentiation (9).

To better understand the fiber–microbiome relationship, 16S ribosomal RNA (rRNA) gene amplicon sequencing has been widely used to assess compositional shifts in fecal samples following RS intervention (10–16). However, findings from these individual studies remain inconsistent. In terms of microbial diversity, while some studies showed that RS intervention lowered the alpha diversity (11, 16), others found no detectable difference between baseline and post-intervention states (15). Similarly, results regarding beta diversity were also contradictory. For instance, DeMartino et al. (17) observed no significant shifts in community structure following intervention; conversely, Baxter et al. (15) reported significant alterations in the gut microbiome. Moreover, the identified RS-associated microbes varied across studies. The butyrate producing genus *Faecalibacterium* has been shown to increase after supplementation in some cohorts (11, 16), but was not found to be responsive or even decreased in others (12, 13). Some interventions reported no significant change in *Bifidobacterium* abundance (17, 18) while others observed a marked enrichment of a well-known fiber-fermenting genus, following a similar dietary intervention (16).

Much of this variability probably traces back to methodological differences—particularly the choice of 16S rRNA hypervariable region and bioinformatic pipeline—which can introduce inconsistencies that have no bearing on the underlying biology. Across studies, such technical variation can mask the microbial signature associated with RS. To address this, raw sequencing data from seven public 16S rRNA datasets were reprocessed using a single standardized pipeline, and strict within-individual pairing was applied to account for baseline differences. By integrating multiple cohorts, this study aims to identify gut microbiome reproducibly altered by RS-predominant dietary fiber interventions and to assess their potential roles in host immune regulation. Within this diet–immune axis, the gut immune microenvironment occupies a central position, encompassing epithelial cells, resident immune cells, secretory IgA, and the gut microbiota—all of which contribute to mucosal immune homeostasis (19). This usage is distinct from gut-associated lymphoid tissue (GALT), which denotes organized lymphoid structures, and from intestinal immune homeostasis, a broader term for the maintenance of mucosal immune balance. Commensal species influence this balance in markedly different ways. *Ruminococcus gnavus*, for instance, produces glucorhamnan lipoglycans that activate TLR4 signaling in dendritic cells, driving the pro-inflammatory Th1/Th17 responses associated with Crohn’s disease. (20). In contrast, metabolites derived from saccharolytic fermentation, especially short-chain fatty acids such as butyrate, have been proposed to support epithelial barrier function and favor immunoregulatory pathways, including Foxp3 + Treg differentiation (9, 21, 22). The gut immune microenvironment is governed through pattern recognition receptors (TLRs, NLRP3) and metabolite-sensing receptors (GPR41/GPR43, PPARγ, AhR), collectively shaping mucosal barrier function, macrophage M1/M2 polarization, and Treg/Th17 balance (23–25). Whether RS can shift this immune microenvironment in an immunologically meaningful direction through selective altering of microbial community composition, however, remains to be established.

Given the connections between gut dysbiosis and inflammatory conditions such as inflammatory bowel disease (IBD), identifying whether RS-responsive taxa overlap with disease-associated microbes would provide hypotheses linking dietary fiber intake to mucosal immune regulation. Characterizing this cross-cohort ecological signature may help clarify the microbial basis of RS-associated health effects and its potential as a therapeutic approach in immune-mediated gut disease, while also providing a microbiological basis for precision nutrition strategies targeting individuals with pathobiont-enriched gut communities.

## Materials and methods

### Study cohorts

Cohorts were selected based on the following criteria: (1) profiling the healthy human fecal microbiome using high-throughput 16S rRNA gene amplicon sequencing; (2) providing publicly available raw sequencing data; (3) reporting sufficient clinical metadata; and (4) employing longitudinal sampling with matched baseline and end-point samples during a defined resistant starch (RS) intervention. Ultimately, seven independent studies met the eligibility requirements (PRJEB41443, PRJNA293971, PRJNA306884, PRJNA891951, PRJNA428736, PRJNA560950, and PRJNA780023). The raw 16S rRNA sequencing data were downloaded using the SRA Toolkit (v.2.10.8). Cohort-level metadata were extracted from original publications and associated public records and summarized in Supplementary Table 1; unavailable variables are recorded as not reported.

Given the considerable heterogeneity across included studies in study design, control diet composition, and washout criteria, data from control conditions and washout periods were not incorporated into the pooled analysis. Instead, only strictly paired baseline and end-point samples collected during defined RS exposure were retained for analysis. This strategy was chosen to control for pronounced inter-individual heterogeneity in baseline microbiome composition across cohorts, but it cannot fully account for temporal drift. Accordingly, the pooled estimates should be interpreted as cross-cohort pre/post associations with RS exposure rather than causal effects attributable solely to RS.

### Standardized microbiome analysis pipelines

Raw reads were processed using a uniform bioinformatics pipeline to minimize technical variation. Fixed 5’ trimming was implemented during DADA2, (26) preprocessing with filterAndTrim; trimLeft = 24 was applied to all cohorts. And, then all cohorts were denoised as forward reads only with truncLen = 150, truncQ = 0, and rm.phix = TRUE, and sequences shorter than 50 nt after filtering were discarded. Chimeras were removed within DADA2 using removeBimeraDenovo (method = “consensus”). Given that the included studies targeted different hypervariable regions of the 16S rRNA gene, closed-reference clustering was performed at 99% sequence identity against the Greengenes2 database (v 2022.10) (27) using the QIIME 2 vsearch plugin (28). This crucial step effectively converted the region-specific ASVs into standardized Operational Taxonomic Units (OTUs), yielding a harmonized cross-cohort abundance table for all downstream analyses. Taxonomic labels for harmonized reference OTUs are based on Greengenes2 annotations and should be regarded as putative, particularly given that datasets targeted different 16S hypervariable regions. Cohort-level DADA2 retention, ASV match rates, and read retention after closed-reference mapping are provided in Supplementary Table 2. Although ASV match rates varied across cohorts, read retention remained high in all studies, though some region-specific bias may remain.

### Microbial diversity analyses

Samples with fewer than 1,000 OTU sequences were excluded from downstream analyses. For alpha and beta diversity analyses only, all samples were rarefied to a depth of 1,000 reads per sample to maximize the retention of paired intra-individual samples across diverse sequencing cohorts. Alpha diversity metrics, including Shannon index, Observed OTUs, and Faith’s phylogenetic diversity (Faith’s PD), were calculated using QIIME2 *q2-diversity* plugin. Faith’s phylogenetic diversity was calculated from the Greengenes2 reference tree. First, the statistical significance of the longitudinal shift within each individual dataset was assessed using a paired Student’s *t*-test. The standardized mean difference (Hedges’ g) (29) and its variance were calculated to quantify within-individual changes between baseline and end-point. Study-specific effect sizes were then pooled using a random-effects model. The pooled effect and its statistical significance were estimated using the Restricted Maximum Likelihood (REML) approach, which accounts for between-study heterogeneity (implemented via the rma function in the metafor R package).

Beta diversity was assessed using Bray–Curtis, Jaccard, and weighted and unweighted UniFrac distance matrices. Principal coordinate analysis (PCoA) and PERMANOVA (adonis2 in the vegan R package, 999 permutations) (30) were first applied to baseline samples to evaluate global community structure and the variance explained by study. To account for substantial between-individual heterogeneity at baseline, a paired design was used and a repeated-measures PERMANOVA (distance ∼ group) was performed, with permutations restricted within subjects [how(blocks = subject_id)]. This approach was applied to each cohort to obtain study-specific marginal *R*^2^ and *p*-values, and to the combined dataset for an overall analysis.

Finally, to compare intervention efficacy across different RS types (RS1, RS2, and RS4), the intra-individual beta diversity distance (baseline vs. end-point) was extracted for each subject. These “paired distances” served as a direct proxy for the magnitude of microbiome restructuring and were evaluated across RS categories using pairwise Wilcoxon rank-sum tests, with Benjamini-Hochberg false discovery rate (FDR) correction.

### Identification of differentially abundant taxa across cohorts

To identify microbial OTUs responding to RS interventions, a paired differential abundance analysis was conducted. To account for the compositional nature of microbiome data, count matrices were first normalized within each cohort using a centered log-ratio (CLR) transformation following established methods (31).

The magnitude of the RS effect was quantified as the intra-individual difference between paired CLR-transformed endpoint and baseline abundances (ΔCLR = CLR_endpoint - CLR_baseline), which is referred to throughout the manuscript as ΔCLR. A pseudocount of 1 was added to all count values before CLR transformation to handle structural zeros. OTUs detected in fewer than 10% of samples were excluded from the differential abundance analysis. To critically evaluate the significance of these shifts across the integrated datasets while accounting for cohort-specific batch effects, a linear mixed-effects model (LMM) of the form ΔCLR ∼ 1 + (1 | study) was build, where study was assigned as a random intercept to capture between-cohort variability in baseline composition and intervention conditions. Subject-level pairing was represented by computing the within-subject difference (ΔCLR) before modeling, so each observation in the LMM corresponds to each participant’s change from baseline to post-intervention. Finally, *p*-values were adjusted by the Benjamini-Hochberg false discovery rate (FDR), and taxa with an FDR < 0.05 were considered significantly differentially taxa.

### Prediction of metagenomic functions

The PICRUSt2 algorithm was employed to predict metagenomic functional potential from the OTU abundance table, inferring the relative abundances of Kyoto Encyclopedia of Genes and Genomes (KEGG) Orthologs (KOs) and Carbohydrate-Active enZymes (CAZymes) (32). Because these functions were inferred rather than directly measured, all downstream interpretations were treated as predictive and hypothesis-generating. Mean ± SD NSTI values were low across all cohorts: 0.0433 ± 0.0093 for PRJEB41443, 0.0480 ± 0.0123 for PRJNA293971, 0.0440 ± 0.0090 for PRJNA306884, 0.0381 ± 0.0113 for PRJNA428736, 0.0395 ± 0.0089 for PRJNA560950, 0.0458 ± 0.0138 for PRJNA780023, and 0.0532 ± 0.0078 for PRJNA891951. All cohort means were below the commonly used reliability threshold of 0.15, supporting the overall robustness of the PICRUSt2-based functional predictions in this dataset. To identify significant differences in the predicted functional profiles, the same statistical methodology used for the taxonomic differential taxa analysis was applied.

### Machine learning analysis

Classification modeling was performed using the Random Forest algorithm implemented via the scikit-learn library (n_estimators = 500, with all other parameters set to their defaults) in Python (33) to discriminate between baseline and post-intervention (end-point) states using taxonomic and functional features. Performance was quantified via Area Under the Receiver Operating Characteristic Curve (AUC) scores. We implemented three distinct validation strategies to ensure the reliability of our models. Model performance was first evaluated within each study using 5-fold cross-validation (within-study CV). Folds were defined at the participant level, such that baseline and end-point samples from the same individual were assigned exclusively to either the training or testing set. This design avoids subject-level feature leakage and ensures that the resulting AUC reflects discrimination of intervention state rather than individual identity. To assess batch effects and model transferability, we performed cross-study validation (CSV), in which models trained on one dataset were evaluated on all other independent datasets. We further applied leave-one-study-out (LOSO) validation, training the classifier on all but one study and testing on the held-out study to identify signals that generalize across cohorts.

### Network analysis

To examine how RS reshapes microbial network structure, the co-occurrence networks were constructed for baseline and end-point samples separately. Pairwise microbial associations were inferred using SparCC (34). Networks from individual studies were then integrated using NetMoss2 (35). Edges were retained at | *r*| > = 0.1 and permutation-based *p* < 0.05. Negative correlations were kept as a separate edge class and visualized separately from positive correlations. Pairwise correlations were calculated only for OTU pairs observed in at least 10% of samples. For cross-cohort integration, NetMoss2 was used to calculate node-level rewiring scores within each cohort, and these scores were then combined across cohorts to rank taxa by the consistency of their topology changes. Networks were visualized in Cytoscape (36).

### Cross-disease validation in inflammatory bowel disease

To explore the clinical relevance of RS-depleted taxa, the abundance of these taxa in inflammatory bowel disease (IBD), a condition characterized by gut microbial dysbiosis was analyzed. Eight publicly available 16S rRNA amplicon sequencing datasets were assembled (Table 1), including PRJNA324147, PRJNA368966, PRJNA422193, PRJNA431126, PRJNA450340, RISK_PRISM_f, qiita_1629, and qiita_2538. These datasets cover 2,491 fecal samples from healthy controls (HC) and patients with Crohn’s disease (CD) or ulcerative colitis (UC). Raw sequences were processed using the pipeline described above. For enrichment analysis, the data were transformed to relative abundances. For each sample, a module-level score was obtained by summing the relative abundances of 22 RS-depleted OTUs (FDR < 0.05). Group differences were assessed using a linear mixed-effects model (LMM), with study treated as a random effect [module_abundance ∼ diagnosis + (1 | study)], followed by pairwise comparisons performed with the emmeans R package and FDR correction. The same modeling framework was also applied to *Ruminococcus gnavus*, based on its sensitivity to RS and its reported link to IBD.

**TABLE 1:** Summary of IBD cohorts used for cross-disease validation.

Study ID	Country	16S region	n (HC)	n (CD)	n (UC)	Total
PRJNA324147	USA	V4	53	25	10	88
PRJNA368966	Prague	V3–V4	29	–	32	61
PRJNA422193	Spain	V4	34	34	39	107
PRJNA431126	China	V4	34	185	97	316
PRJNA450340	Canada	V4	45	42	39	126
RISK_PRISM_f	USA	V4	65	362	105	532
qiita_1629	Sweden	V4	55	211	286	552
qiita_2538	USA	V4	360	349	–	709
Total			675	1,208	608	2,491

HC, healthy control; CD, Crohn’s disease; UC, ulcerative colitis; –, not available in this cohort. PRJNA368966 was included only in the HC vs. UC analysis; qiita_2538 was included only in the HC vs. CD analysis.

To assess whether RS-depleted taxa retain discriminative signal independent of the broader microbiome, random forest (RF) classifiers were trained using a leave-one-study-out (LOSO) approach for HC vs. CD and HC vs. UC comparisons. Models were built using either the full OTU table or the 22 RS-depleted OTUs as features. Performance was evaluated using the area under the curve (AUC), with differences between feature sets assessed by paired Wilcoxon signed-rank tests across cohorts.

## Results

### Study characteristics

**FIGURE 1:**
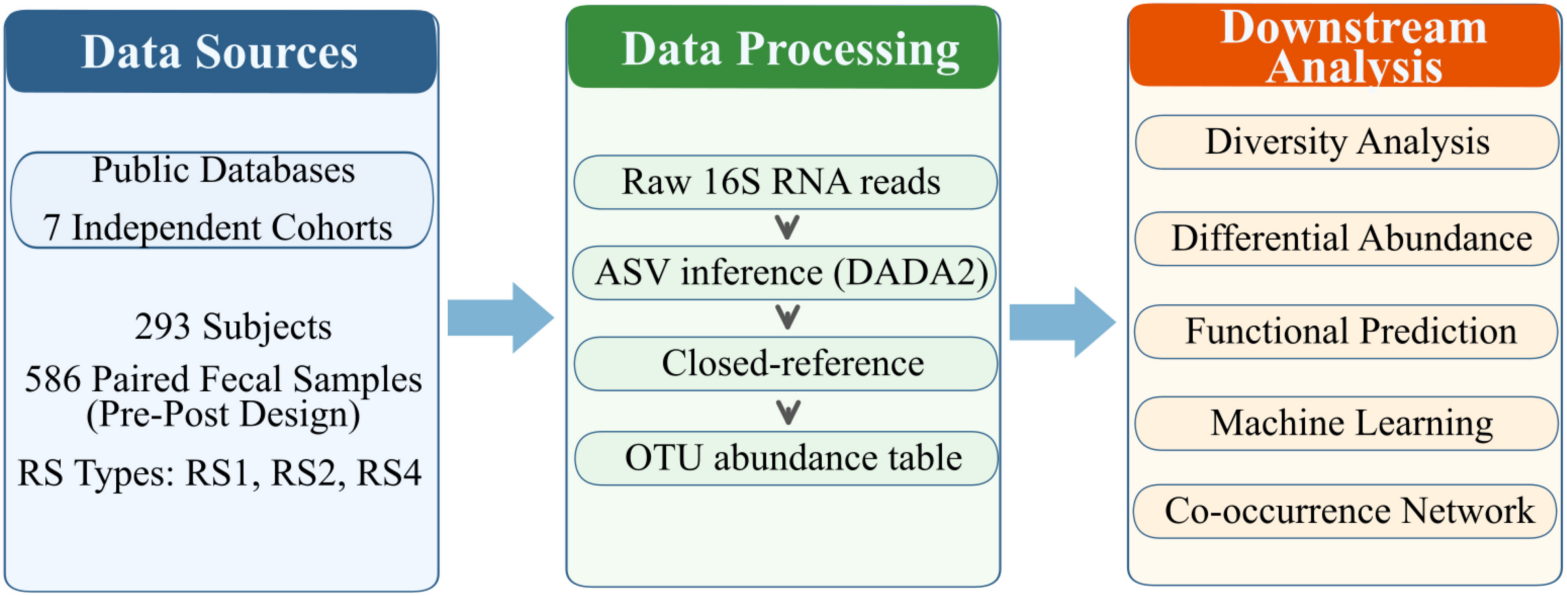
Study design and analytical workflow. Seven independent 16S rRNA amplicon sequencing datasets were processed using a unified bioinformatics pipeline, followed by analyses to characterize gut microbiota responses to resistant starch across cohorts.

Following quality filtering, seven publicly available 16S rRNA amplicon sequencing datasets from healthy human gut microbiome studies were integrated (Table 2; see Methods). Samples from multiple intervention arms, control groups, and washout periods were included in the initial dataset. To reduce methodological heterogeneity across studies with cross-over and parallel designs, a standardized within-individual pairing strategy was used. Only strictly paired baseline and end-point samples from defined resistant starch (RS) intervention periods were retained. A total of 293 healthy subjects and 586 paired samples were included in the final dataset (Figure 1 and Table 2). The cohort-level availability of host and sampling metadata is summarized in Supplementary Table 1.

**TABLE 2:** Fecal 16S studies of fiber intervention in this meta-analysis.

Accession no.	Article info.	Original design	Extracted arm/period	Fiber type and dose	Sequencing region	Analyzed subjects (N)	Paired samples (baseline/end-point)	Country
PRJEB41443	Riley_2021	Crossover	RS period	RS2 (Wheat), 14–19 g/d, 1 week	V3V4	30	60 (30/30)	USA
PRJNA293971	Kovatcheva_2015	Crossover	BKB period	RS1 (Barley), 3 days	V1V2	20	40 (20/20)	Sweden
PRJNA306884	Venkataraman_2016	Pre-post design	Whole cohort	RS2 (Potato), 24 g/d, 7 days	V4	20	40 (20/20)	USA
PRJNA891951	Dahl_2016	Crossover	All RS4 periods	RS4 (Potato, chemically modified potato), 30 g/d, 2 weeks	V1V2	57	114 (57/57)	USA
PRJNA428736	Baxter_2019	Parallel	RS group	RS2 (Potato), 28–34 g/d and RS2 (Maize), 20–24 g/d, 2 weeks	V4	86	172 (86/86)	USA
PRJNA560950	Deehan_2020	Parallel	All RS4 groups	RS4 (Maize/Potato/Tapioca, chemically modified potato), 10–50 g/d, 4 weeks	V5V6	30	60 (30/30)	Canada
PRJNA780023	Peter_2022	Crossover	Potato (POT) period	RS2(Potato), 2.4 g/d, 4 weeks	V4	50	100 (50/50)	USA

All included studies followed a within-subject pre–post design without parallel control group. Therefore, the final dataset comprised harmonized, within-individual pre/post comparisons during defined RS intervention periods drawn from pooled cross-cohort data, and the results should be interpreted as associations rather than causal effects. Healthy adults were included in the cohorts, primarily from North America (five from the USA and one from Canada) and Europe (one from Sweden). A range of RS types was used in the interventions, including physically inaccessible RS1 (barley), granular RS2 (wheat, potato, and maize), and chemically modified RS4 (potato, maize, and tapioca). Dosages ranged from dietary intakes (∼2.4 g/day) to high-dose supplementation (up to 50 g/day), with intervention durations spanning 3 days to 4 weeks. Multiple 16S rRNA hypervariable regions were sequenced, including V1–V2, V3–V4, V4, and V5–V6.

### RS acts as a selective ecological filter: cross-cohort reduction in diversity without broad community disruption

**FIGURE 2:**
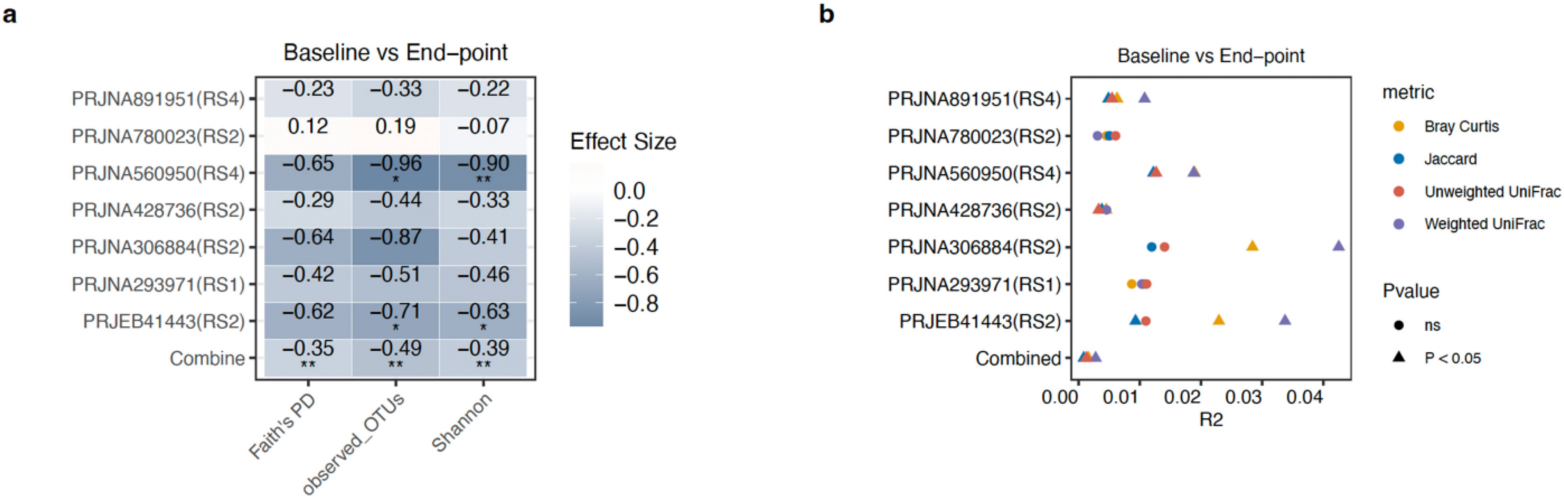
Effect of dietary fiber on gut microbial diversity. **(a)** Heatmap showing differences in alpha diversity between the baseline and end-point states in each study. The “Combined” row represents the pooled effect size calculated using a random-effects model (REM). Blue color indicates decreased diversity following the RS intervention relative to baseline; red color indicated higher diversity end-point. **(b)** Variance (R^2^) explained by the fiber intervention determined by ADONIS2. The combined R^2^ value was calculated using study identity as a stratifying factor to account for inter-study variation. Triangles denote statistical significance (*P* < 0.05). Per-cohort numbers of paired subjects were as follows: PRJEB41443 (*n* = 30); PRJNA293971 (*n* = 20); PRJNA306884 (*n* = 20); PRJNA891951 (*n* = 57); PRJNA428736 (*n* = 86); PRJNA560950 (*n* = 30); PRJNA780023 (*n* = 50); Combined (*n* = 293). *p < 0.05; **p < 0.01.

Alpha diversity was reduced following RS supplementation across all three metric, including Faith’s phylogenetic diversity (PD), observed OTUs, and Shannon entropy, relative to baseline (pooled Hedges’ g: -0.35, -0.49, and -0.39, respectively; all *p* < 0.05; Figure 2a). This result suggests that RS may function as a selective ecological filter. Community diversity may be reduced as taxa capable of efficiently fermenting RS are selectively enriched. That said, one dataset (PRJNA780023) showed a modest diversity increase, which likely reflects cohort-specific differences in baseline microbiome composition, or perhaps the relatively low RS dose employed in that study (2.4 g/day). Either way, this observation does not materially change the broader interpretation.

**FIGURE 3:**
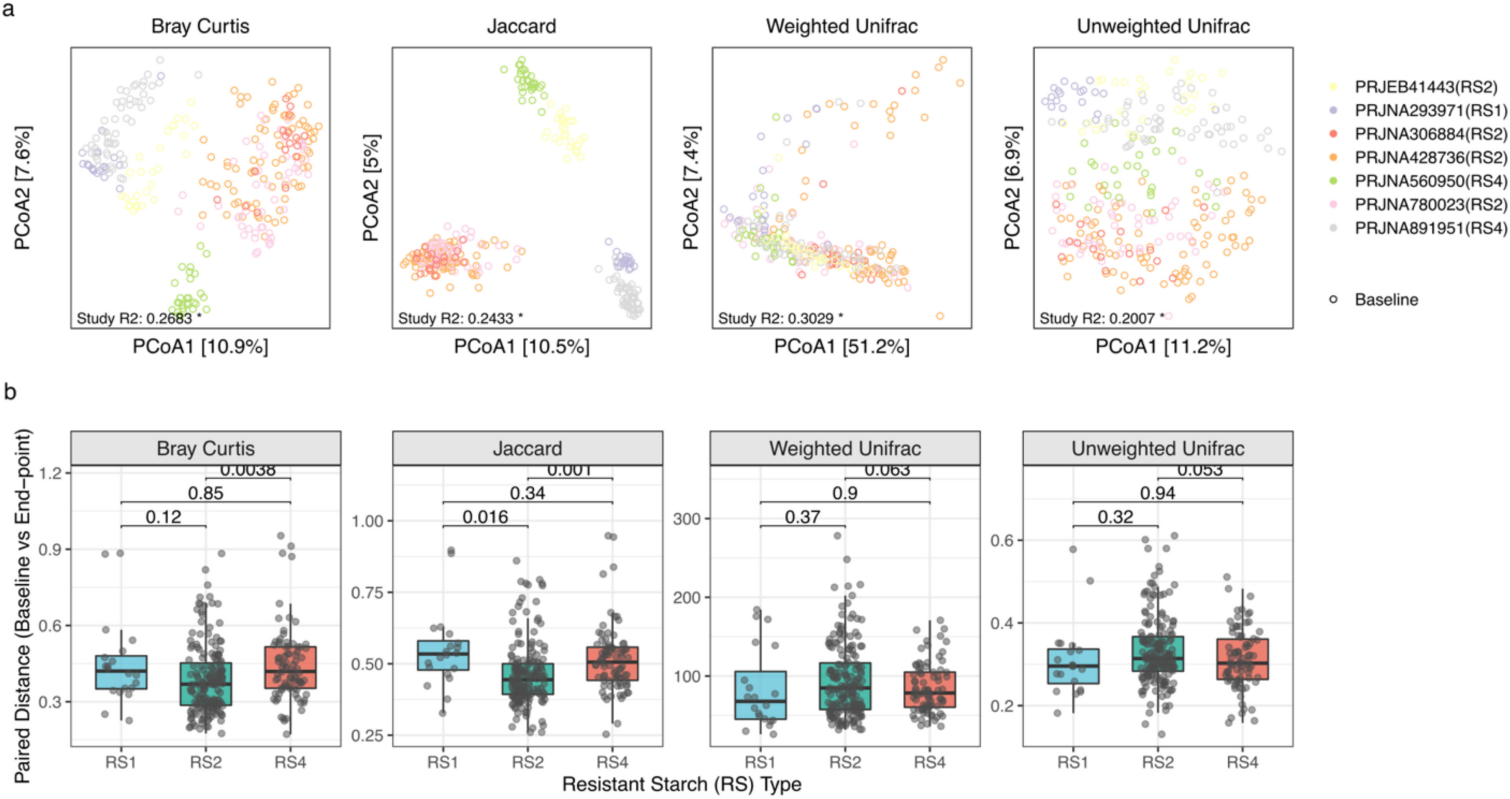
Baseline cohort heterogeneity and the magnitude of microbial community reorganization driven by different resistant starch (RS) types. **(a)** Global principal coordinates analysis (PCoA) of gut microbiota profiles showed distinct clustering by study origin across four distance metrics: Bray–Curtis, Jaccard, weighted UniFrac, and unweighted UniFrac. Each point represents a sample, colored by study cohort. The variance explained by study cohort (Study R^2^) is shown at the bottom of each panel, reflecting baseline differences between cohorts. **(b)** Boxplots of intra-individual paired beta-diversity distances (baseline versus end-point), stratified by RS structural class (RS1, RS2, and RS4). This paired metric captures the level of structural change within each subject while accounting for baseline variation. *P*-values for comparisons between RS groups are shown above the corresponding brackets.

Beta diversity analyses further confirmed that RS-associated community shifts reflected changes in relative abundance rather than outright species replacement. The magnitude of RS-induced gut microbial community change varied considerably across studies, with the most pronounced shifts observed in PRJNA891951, PRJNA560950, and PRJNA428736, but only marginal (*p* < 0.05) or non-significant (*p* > 0.05) changes in the remaining cohorts (Figure 2b). Integrated analysis revealed a consistent global effect across all dissimilarity metrics, including Bray-Curtis, Weighted UniFrac, Unweighted UniFrac, and Jaccard. Notably, and effect sizes were systematically larger for abundance-based metrics than for presence-absence metrics, suggesting that RS reshapes community composition primarily by altering the relative abundances of resident taxa rather than driving taxonomic turnover. PERMANOVA on baseline samples showed clear study-level clustering, with cohort identity accounting for 20.07–30.29% of variance across all distance metrics (Figure 3a); intra-individual paired distances were therefore used to estimate intervention effects (Figure 3b). High-dose RS4 interventions were associated with larger non-phylogenetic community shifts than lower-dose RS2 regimens (Figure 3b). This pattern likely arises from differences in substrate load, rather than from starch chemistry alone, since RS type and dose were strongly confounded across trials.

### RS consistently depletes a discrete pathobiont-enriched module while failing to universally enrich any beneficial taxon

**FIGURE 4:**
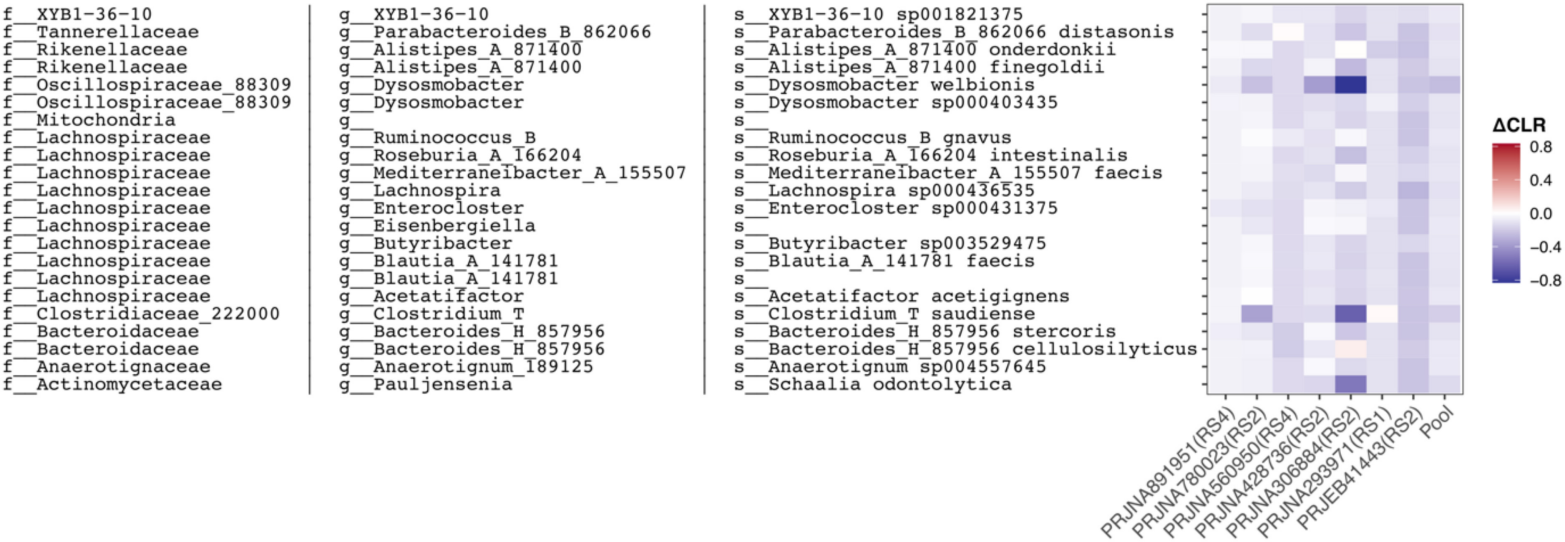
Specific gut OTUs are consistently suppressed following resistant starch intervention. Heatmap of significantly depleted species across seven independent studies. Color intensity represents the mean ΔCLR of each species within each cohort, indicating a consistent negative shift (blue) across studies.

To capture the broader ecological response of the healthy gut microbiota to RS, we examined how individual microbial abundances shifted within subjects across the all cohorts. A general decline in overall microbial abundance was an apparent pattern that mirrored the drop in alpha diversity already observed. Differential abundance analysis reinforced this trend, revealing a strikingly unidirectional response: all 22 taxa passing the significance threshold (FDR < 0.05) were exclusively depleted following the intervention (Figure 4 and Supplementary Table 3), under the current analytical conditions. Further taxonomic examination revealed that several of these consistently depleted microbes are well-known opportunistic pathogens or pro-inflammatory pathobiont. These include putative *Mediterraneibacter faecis*, *Ruminococcus gnavus* [a pathobiont frequently associated with mucosal inflammation and inflammatory bowel diseases (IBD) (20, 37)], and *Schaalia odontolytica* [an oral microbe indicative of ectopic gut colonization that has been implicated in colorectal neoplasia and gut mucosal dysplasia (38)].

The complete absence of universally enriched OTUs highlights profound inter-cohort variability, indicating that the specific primary degraders responding to RS are highly population-specific. The observed taxonomic inconsistency suggests that RS degradation is carried out by a functionally redundant yet taxonomically diverse microbial consortium. It was therefore examined whether functional profiles were more consistent across cohorts than taxonomic composition.

This result of selective pathobiont depletion without universal enrichment of a single beneficial taxon carries direct implications for precision nutrition. Rather than seeking a universal prebiotic responder, future dietary strategies may be better designed around suppressing a defined set of immunostimulatory taxa in high-risk individuals. To this end, the 22-OTU module identified here represents a candidate microbial signature for stratifying individuals and guiding targeted fiber-based interventions.

### Functional redundancy preserves community metabolic capacity despite targeted taxonomic depletion

To determine whether RS-associated taxonomic shifts were accompanied by alterations in predicted functional capacity, the same paired analysis framework was applied to inferred functional profiles.

**FIGURE 5:**
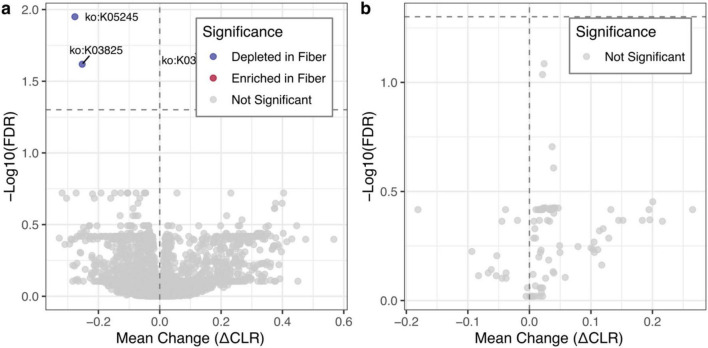
Functional redundancy and broadly consistent predicted metagenomic shifts following resistant starch intervention. **(a)** Volcano plot of differentially abundant KEGG Orthology (KO). The x-axis shows the mean intra-individual change (ΔCLR), while the y-axis represents the -log10-transformed false discovery rate (FDR). The overall distribution is centered near zero, with only three KOs significant (FDR < 0.05): one enriched (red) and two depleted (blue). K05245: L-carnitine/gamma-butyrobetaine antiporter; K03825: L-phenylalanine/L-methionine N-acetyltransferase; K03316: Na + :H + antiporter. **(b)** Volcano plot illustrating the differential abundance of Carbohydrate-Active enZymes (CAZymes). Although the global distribution exhibits a general shift toward positive ΔCLR values (indicating widespread mild increases), no individual CAZyme family achieved statistical significance (FDR < 0.05).

Fold changes across KEGG Orthology (KO) terms were generally centered around zero. Only three KOs were significant (FDR < 0.05), including one enriched (K03316) and two depleted (K05245 and K03825) after the intervention (Figure 5a). Functional changes were limited, but the identities of these KOs may still shed light on microbial responses to RS. The enriched term (K03316) encodes a Na^+^:H^+^ antiporter involved in intracellular pH regulation. The increase may reflect adaptation to shifts in the luminal environment, potentially linked to short-chain fatty acid (SCFA) production during fermentation (39, 40). The two depleted KOs (K05245 and K03825) correspond to an L-carnitine/γ-butyrobetaine antiporter and an L-phenylalanine/L-methionine N-acetyltransferase, respectively. These reductions may reflect shifts in pathways related to carnitine transport and amino acid metabolism. This result suggests a shift toward saccharolytic activity over proteolytic metabolism, although it will require further experimental validation.

Because resistant starch is a complex polysaccharide, we next examined Carbohydrate-Active enZymes (CAZy) annotations (41). CAZyme profiles showed a shift toward positive values (ΔCLR > 0) (Figure 5b), suggesting an overall increase in carbohydrate-related functional potential. However, no individual CAZyme family remained significant after multiple testing correction. Together, these findings suggest that although RS is associated with the depletion of specific microbial OTUs, the overall metabolic structure of the gut microbiome remains relatively stable. This functional stability has important implications for precision nutrition: it indicates that interventions targeting this pathobiont module are unlikely to destabilize core community metabolism, supporting the safety and tractability of RS as a dietary tool for modulating immune-relevant microbial composition without broadly disrupting gut function.

### Taxonomic and function profiles yield comparable cross-cohort predictive performance, while CAZyme signals remain modest

Although functional profiles remained largely stable, the consistency of the taxonomic shifts suggests that RS induces a reproducible compositional pattern not captured by any single feature. To assess whether this signal generalized across cohorts, we applied random forest (RF) classifiers to predict post-intervention status using taxonomic (OTU), functional (KO), and CAZyme profiles. Model performance was evaluated using within-study cross-validation (CV), cross-study validation (CSV), and leave-one-study-out (LOSO) validation.

**FIGURE 6:**
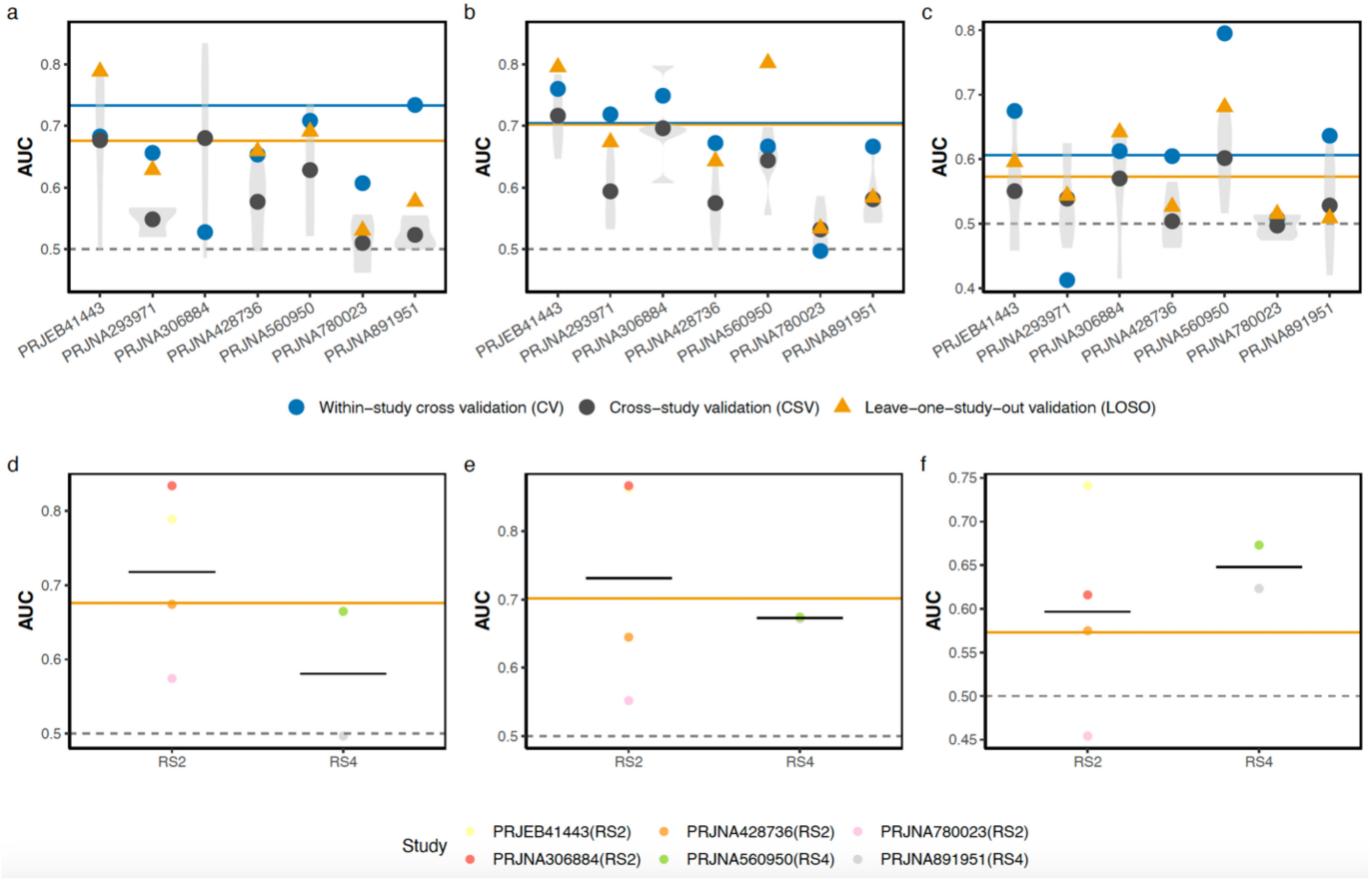
Discrimination of intervention state of gut microbiome and functional potentials for resistant starch intake within and across cohorts. **(a–c)** Area under the ROC curve (AUC) values of Random Forest (RF) models based on **(a)** taxonomic (OTU), **(b)** KEGG Orthology (KO), and **(c)** Carbohydrate-Active enZymes (CAZyme) profiles. Violin plots depict the distribution of cross-study validation (CSV) performance, where study-specific models were tested on data from every other study, with AUCs reported for each test set along the x-axes. Gray dots represent the average CSV AUC for each study. Blue circles and yellow triangles indicate AUCs for within-study cross-validation (CV) and leave-one-study-out (LOSO) validation, respectively. Horizontal lines denote the average AUC across all seven cohorts for the corresponding validation strategy (blue line for CV; yellow line for LOSO). LOSO AUCs were obtained by training models on an integrated dataset excluding one specific study, which was subsequently used as the independent hold-out test set. **(d–f)** Exploratory predictive performance (AUC) of LOSO validation models stratified by resistant starch structural classes (RS2 and RS4) for **(d)** taxonomic (OTU), **(e)** KO, and **(f)** CAZyme profiles. RS1 was excluded from this sub-analysis as it was represented by only a single cohort. Horizontal black lines indicate the mean AUC within each RS subgroup, with individual colored points representing the hold-out test performance for specific study cohorts.

Taxonomic profiles achieved a within-study CV AUC of ∼0.73 (Figure 6a), indicating a consistent response to RS within individual cohorts. Performance declined under cross-study validation (CSV) and showed greater variability, consistent with baseline differences across cohorts. In contrast, leave-one-study-out (LOSO) validation yielded a more stable AUC of 0.68. This suggests that combining datasets can reduce cohort-specific variation and reveal signals that are shared across studies.

Functional profiles showed a complementary pattern. KO-based models (Figure 6b) generalized well across cohorts, with LOSO performance comparable to within-study CV (average AUC ∼0.70). This consistency is in line with the functional redundancy observed above, where metabolic pathways appear more stable across datasets than taxonomic features. CAZyme-based models (Figure 6c) were also predictive but showed lower performance overall (CV AUC ∼0.61; LOSO AUC ∼0.58), consistent with the relatively modest and distributed changes observed in carbohydrate-active enzyme families. Stratifying LOSO models by RS type (Figures 6d–f; RS1 cohort excluded) revealed distinct patterns that may relate to the dose–starch effects observed in the beta-diversity analysis. Models trained on RS2 datasets showed consistent improvements across feature types, with taxonomic performance reaching an AUC of ∼0.72. In contrast, RS4 models showed lower performance for taxonomic and KO features but yielded the strongest CAZyme-based signal. These differences may reflect variation in substrate exposure, as RS4 interventions generally involved higher doses (up to 50 g/day). Such conditions may preferentially amplify enzymatic responses compared with more moderate RS2 regimens, independent of starch type. The RS4 LOSO analyses were considered exploratory because only two cohorts were available for training and testing.

Collectively, these classification results confirm that the RS-associated ecological state characterized by reduced diversity, uniform taxonomic depletion, and functional homeostasis, constitutes a reproducible, cross-cohort signature, most stably captured by functional rather than taxonomic profiles. Overall, these results show that RS intervention is associated with a reproducible microbial signature across cohorts. This signal was detected more consistently from functional profiles than from taxonomic profiles. However, because the classifiers were trained to distinguish baseline from end-point samples, these analyses indicate discrimination of intervention state rather than prospective prediction of individual responsiveness from baseline microbiota.

### Indirect network modulation underlies pathobiont marginalization following RS intervention

**FIGURE 7:**
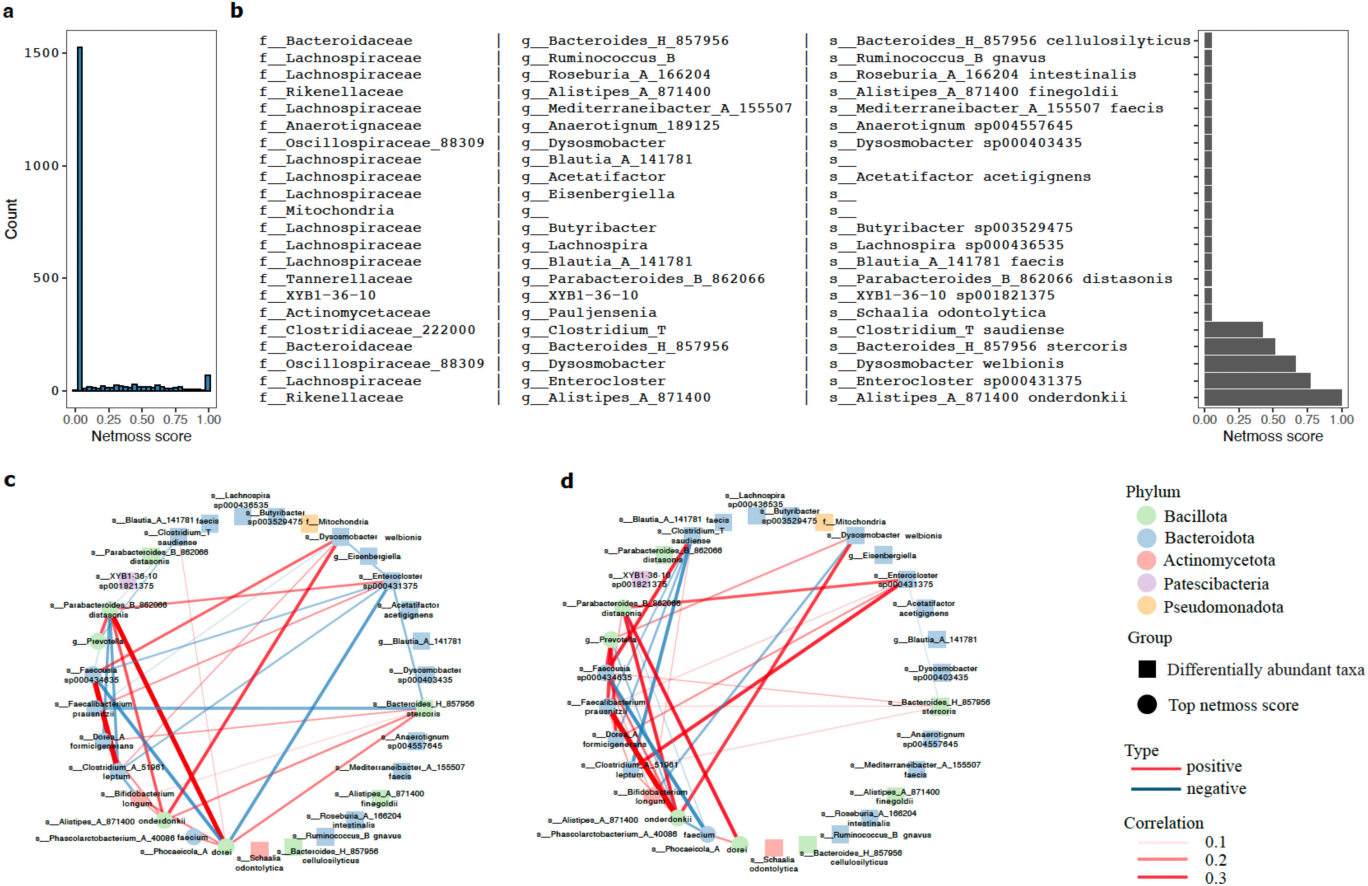
Topological rewiring and niche isolation within the gut microbiome driven by resistant starch intervention. **(a)** Histogram showing the distribution of NetMoss scores across the global microbial community. The vast majority of taxa score near 0, indicating a largely stable overall network framework, while only a select few (scores approaching 1) underwent drastic topological rewiring. **(b)** NetMoss score distribution for the differentially abundant taxa (microbes significantly depleted following RS intervention). With a single exception, the scores of these RS-sensitive taxa fall almost exclusively within the lowest range. **(c,d)** Core-periphery sub-network visualizations at **(c)** baseline and **(d)** end-point. Circular nodes represent the top 10 core driver microbes (highest relative abundance among those with a NetMoss score of 1), while square nodes represent the taxa significantly depleted in the differential abundance analysis. Node colors denote their corresponding phyla. Edges indicate positive (red) or negative (blue) correlations, with line thickness proportional to the absolute strength of the correlation coefficient.

To explore the ecological basis of the observed depletion pattern, the NetMoss2 algorithm (35) was used to assess intervention-associated changes in co-occurrence network topology (Figure 7a). Network reorganization was limited to a small subset of OTUs with high NetMoss scores (>0.12), whereas most taxa showed little change in their interaction structure. Notably, RS-sensitive taxa identified in Figure 4, including opportunistic pathogens such as *Ruminococcus gnavus* and co-depleted commensal taxa, had consistently low NetMoss scores (Figure 7b), suggesting that they were not major contributors to network restructuring after RS intervention. This pattern indicates that the depletion of taxa may reflect indirect ecological shifts rather than direct effects of RS.

To visualize these changes, comparative sub-networks were constructed at baseline and at the end of the intervention, incorporating the top 10 driver OTUs (circular nodes) alongside significantly depleted taxa (square nodes) (Figures 7c,d). At baseline, several taxa that were later depleted were positively associated with the broader community. After RS intervention, driver OTUs, including *Bifidobacterium longum*, *Phocaeicola dorei*, *Prevotella* spp., *Faecalibacterium prausnitzii*, and *Clostridium leptum*, formed a more interconnected core, whereas RS-sensitive taxa shifted toward the network periphery with fewer connections to the core. This pattern is consistent with a contraction of available niches, in which taxa adapted to carbohydrate fermentation become more central, while less-adapted taxa decline in both connectivity and relative abundance. It bears noting that these network edges reflect statistical co-occurrence rather than verified biological interactions; whether metabolic coupling within this core module is real will need to be tested by meta-transcriptomics or stable isotope probing. The depletion of these taxa most plausibly reflects indirect competitive exclusion rather than direct substrate inhibition—pointing to the possibility that sustained high-fiber intake alone may be sufficient to reshape network topology and durably suppress immune-relevant taxa. If so, the implications for precision nutrition are considerable: continuous dietary RS provision, without pharmacological intervention, may be enough to achieve meaningful modulation of the gut’s immune-relevant microbial composition.

### RS-depleted pathobionts are preferentially enriched in IBD across cross-sectional cohorts

**FIGURE 8:**
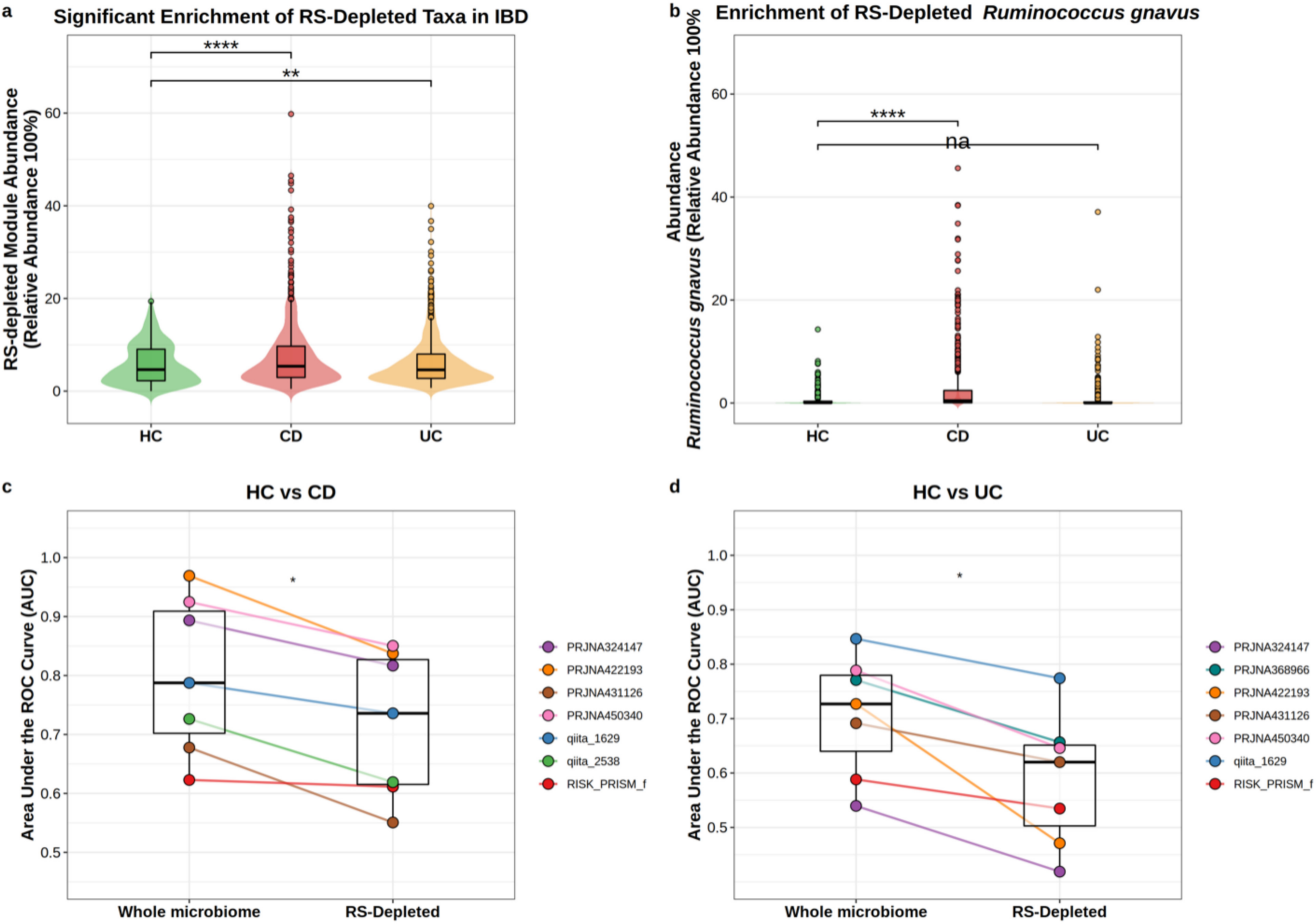
RS-depleted taxa are preferentially enriched in Crohn’s disease and retain cross-cohort discriminative capacity. **(a,b)** Violin plots showing the abundance of the RS-depleted module (sum of 22 RS-depleted OTUs; **a**) and *Ruminococcus gnavus* specifically **(b)** across healthy controls (HC), Crohn’s disease (CD), and ulcerative colitis (UC) in eight independent IBD cohorts (*n* = 2,004). Significance was assessed by linear mixed-effects models with FDR-corrected pairwise contrasts (*p* < 0.0001; *p* < 0.01; ns, not significant). **(c,d)** Leave-one-study-out Random Forest AUC for HC vs. CD **(c)** and HC vs. UC **(d)**, comparing the whole microbiome against the 22 RS-depleted OTUs. Connected lines link paired cohort results; the whole microbiome outperformed the RS-depleted subset across all cohorts (paired Wilcoxon, *p* < 0.05). Treatment exposure varied across cohorts: some studies included patients on immunosuppressants, biologics, or antibiotics, while others excluded recent antibiotic users or provided insufficient detail for direct adjustment. *p < 0.05; **p < 0.01; ***p < 0.001; ****p < 0.0001.

Given that the consistently depleted taxa include established pathobionts, it was subsequently investigated whether these RS-depleted microbes are also enriched in IBD. Having identified a set of microbes consistently suppressed under RS, it was next examined whether these same taxa are elevated in IBD, where gut microbial dysbiosis is well documented (37) and dietary fiber intake is commonly reduced (42). Integrating eight independent IBD cohorts (*n* = 2,491 samples; Table 1), we computed a composite abundance for the RS-depleted module. These IBD datasets were cross-sectional and lacked harmonized information on medication use, antibiotics, diet, and disease activity, so the findings should be treated as hypothesis-generating rather than grounds for clinical translation. Treatment exposure also differed substantially across cohorts: some included patients on immunosuppressants, biologics, or antibiotics, while others excluded recent antibiotic users or simply provided insufficient detail to permit direct adjustment. Patients with CD showed significantly higher module abundances than HC (adjusted mean: 7.35% vs. 5.43%; *p* < 0.0001), and a more modest but significant elevation was also present in UC (adjusted mean: 6.64%; *p* = 0.0048 vs. HC). Module abundance in CD exceeded that in UC, though this difference fell short of statistical significance (*p* = 0.057) (Figure 8a).

At the individual taxon level, the enrichment pattern was far more disease-specific. Of the 22 RS-depleted OTUs, R. gnavus stood out for its selective and substantial elevation in CD (adjusted mean: 2.13% relative abundance) relative to HC (0.66%; *p* < 0.0001, Cohen’s *d* = 0.82), while UC patients showed no significant increase (0.89%; *p* = 0.34) (Figure 8b). That CD and UC diverge in this way, even as both conditions show elevation of the broader RS-depleted module, suggests that the taxa captured by this analysis are not uniformly enriched across IBD subtypes.

We then tested whether this enrichment translates into discriminative signal. LOSO RF models restricted to the 22 RS-depleted OTUs achieved a median AUC of 0.74 across seven independent CD cohorts, compared to 0.79 for the whole microbiome (Figure 8c). For UC, the gap was wider: RS-depleted taxa yielded a median AUC of 0.62 versus 0.73 for the whole microbiome (Figure 8d). The whole microbiome outperformed the RS-depleted subset in all cohorts for both comparisons (paired Wilcoxon, *p* < 0.05), yet the RS-depleted taxa maintained substantial discriminative capacity for CD, with several individual cohorts exceeding an AUC of 0.80. Together, these findings provide hypothesis-generating evidence for potential clinical relevance. Individuals carrying a high baseline abundance of the RS-depleted module may represent a candidate subgroup for precision nutrition interventions with dietary RS. High-risk individuals gut microbiome already resembles the pro-inflammatory dysbiotic profile characteristic of CD, raising the possibility they may derive the greatest immune-modulatory benefit from RS-mediated ecological remodeling.

## Discussion

Results from individual RS studies have been inconsistent, likely because of inter-individual variation and differences in study methodology. Through reprocessing of seven independent cohorts via a unified pipeline and strict intra-individual pairing, a reproducible cross-cohort pre/post microbial pattern associated with RS exposure was identified. Under this design, RS intake was consistently associated with depletion of a defined set of taxa, while the broader predicted functional capacity remained comparatively stable. While these results point to an ecological link between RS intake and a pro-inflammatory microbial shift, attributing all observed changes causally to RS would be premature.

The decrease in alpha diversity following RS intervention is consistent with selective enrichment, as only specific taxa were depleted while the overall community structure remained intact. Beta-diversity analyses support this interpretation: abundance-based metrics (Bray–Curtis, weighted UniFrac) consistently yielded larger effect sizes than presence–absence metrics (Jaccard, unweighted UniFrac), indicating that RS acts primarily on relative abundances rather than species composition. Network analysis adds further nuance. Under RS exposure, saccharolytic degraders and secondary fermenters coalesce into a more interconnected core, while taxa less suited to the substrate are pushed to the periphery. Consistent with this observation, depleted taxa show low NetMoss scores, indicating a greater role for indirect ecological effects than direct responses to RS.

The identity of the depleted taxa suggests potential immunological relevance of this ecological shift. The 22 OTUs consistently reduced across cohorts include putative *Ruminococcus gnavus*, *Mediterraneibacter faecis*, and *Schaalia odontolytica*, taxa that have been associated with pro-inflammatory activity. For example, *R. gnavus* produces a glucorhamnan polysaccharide that can activate TLR4-dependent signaling in dendritic cells, linking its abundance to mucosal immune responses. These observations suggest that RS may act as an ecological filter, reducing taxa with pro-inflammatory potential within the gut immune microenvironment.

Despite these taxonomic changes, predicted functional capacity remained relatively stable, consistent with metabolic redundancy, whereby functionally similar taxa may compensate for those that are depleted. Only three KEGG Orthology terms reached significance, none related to SCFA biosynthesis. As such, potential butyrate-associated immunological effects, including Foxp3^+^ Treg differentiation and GPR109a activation (9, 21, 22), are not directly supported by these data. These functional inferences are based on PICRUSt2 predictions and will require validation using metagenomic and metabolomic approaches. Nevertheless, the overall pattern is consistent with a possible shift toward greater saccharolytic potential and lower putrefactive metabolism. Given that putrefactive fermentation can generate metabolites such as LPS and p-cresol that engage TLR4 and affect epithelial barrier integrity, the observed reduction in pathways related to carnitine transport and amino acid metabolism may indicate a decrease in baseline immunostimulatory signaling within the gut immune microenvironment, although this remains to be confirmed experimentally.

The overlap between RS-depleted taxa and IBD-associated microbiota provides the most direct connection between the ecological findings and gut immune homeostasis. Cross-disease validation across eight IBD cohorts confirmed that the RS-depleted module is significantly elevated in CD and UC. Enrichment was sharpest for *R. gnavus*: elevated in CD but not in UC (*p* = 0.34), consistent with CD’s distinct small-intestinal TLR4-driven immune profile. The RS-depleted subset alone discriminated CD from healthy controls with a median LOSO AUC of 0.74, supporting its candidacy as a diet-linked biomarker panel. Together, these findings indicate that the RS-depleted module captures a pathologically relevant microbial signature connecting dietary RS ecology to a clinically recognized immune-modulatory axis, rather than reflecting generic dysbiosis.

Reverse causation is the principal interpretive threat to these cross-disease findings. Patients with CD routinely restrict dietary fiber due to symptoms and clinical guidance, independently reducing substrate availability for RS-sensitive bacteria and creating a permissive niche for pathobionts, irrespective of any RS protective effect. Without individual dietary intake data or adjustment for medications (immunosuppressants, biologics, antibiotics) and disease activity, the enrichment pattern is equally consistent with disease-driven fiber restriction as with RS-mediated ecological protection. These findings are therefore best treated as hypothesis-generating: they define a testable mechanistic link between dietary RS and gut immune homeostasis, not as evidence that RS would therapeutically suppress these taxa in established IBD.

Machine learning validation confirmed the cross-cohort reproducibility of this ecological footprint. A taxonomic AUC of ∼0.73 was obtained with within-study cross-validation, compared with 0.68 under leave-one-study-out validation, indicating moderate but consistent performance across cohorts. KO-based models showed similar performance under LOSO (AUC ∼0.70), comparable to within-study results, suggesting greater cross-study stability of functional profiles than taxonomic features. Preclinical evidence supports this interpretation. A meta-analysis of 21 animal IBD studies found that RS reduced mucosal damage and increased SCFA production compared with placebo (42). Any apparent differences between RS2- and RS4-stratified models should also be interpreted cautiously, as dose and RS type were strongly confounded across the available cohorts.

Taken together, the findings of this study support a precision-nutrition framework only at the level of hypothesis generation. Our data show that RS consistently reduces a 22-OTU, pathobiont-enriched module across cohorts, and that this module is also enriched in IBD-associated dysbiosis. These observations suggest that baseline abundance of this module may serve as a potential biomarker for inflammatory disease risk in future intervention trials. However, the classifiers developed here distinguish baseline from end-point samples using data from both time points, which is fundamentally different from predicting individual response to RS from baseline data alone. The present study therefore does not support prospective prediction of RS responsiveness at the individual level. A formal validation will require baseline-only responder-prediction models linked to ecological and clinical endpoints. This approach would allow the observed links between dietary fiber and the microbiota to be examined as a clinically relevant hypothesis.

Five methodological limitations apply. First, the analysis relied on harmonized within-individual pre/post comparisons, which reduces baseline inter-individual heterogeneity but cannot fully account for temporal confounding. Control arms and washout periods were excluded, as their definitions, durations, and sampling schemes differed too substantially across studies to permit meaningful harmonization. Second, starch structure and dose are confounded across RS subtypes (RS4: up to 50 g/day), precluding conclusions about structural specificity without dose-matched trials. Third, IBD cohorts lacked individual medication data, preventing adjustment for drug-related microbiome effects in the cross-disease analysis. Fourth, all functional inferences rely on PICRUSt2 predictions; immune consequences remain computational hypotheses without matched metagenomic or metabolomic data. Fifth, all RS intervention cohorts were from North America or Europe, limiting generalizability to non-Western populations.

## Conclusion

By reprocessing raw 16S rRNA data from seven cohorts through a unified pipeline with intra-individual pairing, we identify a cross-cohort pre/post microbial signature associated with RS interventions despite substantial baseline heterogeneity. Across cohorts, RS exposure was consistently associated with depletion of 22 microbial taxa, including the putative TLR4-activating pathobiont *Ruminococcus gnavus* (at OTU resolution), and reproducibly reconfigured co-occurrence networks toward a consolidated saccharolytic core. Predicted functional shifts, expansion of carbohydrate degradation and contraction of putrefactive pathways, suggest an RS-associated ecological recalibration. Downstream immune-modulatory consequences, including effects on Foxp3^+^ Treg differentiation, Th17 expansion, and mucosal barrier integrity, remain speculative. Cross-disease validation confirms that RS- depleted taxa are preferentially enriched in Crohn’s disease, raising the hypothesis that RS modulates a diet-linked immune-relevant microbial module, though reverse causation from fiber restriction cannot be excluded. Evaluating prebiotic associations requires integrating ecological and metabolic evidence; causal conclusions will require parallel-controlled trials with paired metagenomic sequencing, targeted SCFA metabolomics, mucosal immune profiling, and longitudinal sampling across diverse populations. These findings also have implications for precision nutrition. A consistent cross-cohort dietary–microbial footprint comprising 22 taxa depleted by RS supports stratifying individuals by baseline pathobiont module abundance in RS interventions. These findings also have implications for precision nutrition. A consistent cross-cohort dietary-microbial footprint comprising 22 taxa depleted by RS identifies a candidate module suggesting further investigation into its relationship with immune dysregulation. Whether individuals with high baseline abundance of this module derive greater immunological benefit from RS remains unknown and should be tested in prospective, microbiome-informed trials with baseline-only prediction and mucosal immune endpoints.

## Data Availability

The original contributions presented in this study are included in the article/Supplementary material, further inquiries can be directed to the corresponding authors.
