# Supplementary material for: Resistant starch selectively depletes a putative pathobiont-enriched gut microbial module: evidence from multiple dietary fiber intervention cohorts
Source: Front Nutr. 2026 May 20;13:1845191. doi: 10.3389/fnut.2026.1845191 (PMC13230024; doi:10.3389/fnut.2026.1845191)
Supplement: Supplementary file 1 [file Table_1.docx]

**Supplementary Table S1.** Cohort-level availability of host and sampling metadata across the included resistant starch intervention cohorts

| **Study** | **Age reported** | **Sex reported** | **BMI reported** | **Sample collection timing** | **Backgroud diet** | **Antibiotic exclusion** | **Health criteria** |
| --- | --- | --- | --- | --- | --- | --- | --- |
| PRJEB41443 | 40-65 | M=12, F=18 | 26.5 ± 3.8 | 0d, 7d | Free diet + recorded | Not reported | Healthy |
| PRJNA293971 | 50–70 | M=6, F=33 | 18–28 | 0d, 3d | Free diet | Not reported | Healthy |
| PRJNA306884 | 19-20 | M=10, F=10 | 19-63 | 0d, 3d, 4d, 11d | Free diet | Not reported | Healthy |
| PRJNA428736 | 17-29 | Not reported | Not reported | 0d~7d, 14d, 21d | Free diet | Yes | Healthy |
| PRJNA560950 | 28.4 ± 8.1 | M=20, F=20 | 24.0 ± 3.2 | 0d, 7d, 14d, 21d, 28d | Free diet + recorded | Yes | Healthy |
| PRJNA780023 | 40 ± 13 | M=16, F=34 | 24.5 ± 3.6 | 0d, 14d, 28d | Free diet | Not reported | Healthy |
| PRJNA891951 | 18–24 | M=21, F=36 | 18-32 | 0d, 14d, 21d, 35d | Free diet + recorded | Not reported | Healthy |
