# Supplementary material for: Resistant starch selectively depletes a putative pathobiont-enriched gut microbial module: evidence from multiple dietary fiber intervention cohorts
Source: Front Nutr. 2026 May 20;13:1845191. doi: 10.3389/fnut.2026.1845191 (PMC13230024; doi:10.3389/fnut.2026.1845191)
Supplement: Supplementary file 2 [file Table_2.docx]

**Supplementary Table S2.** Cohort-level preprocessing and harmonization metrics, including DADA2 retention and closed-reference read retention.

| **Study** | **DADA2 retained mean pct.** | **DADA2 retained sd pct.** | **ASVs matched to GG2 n** | **ASVs matched to GG2 pct.** | **Reads retained after closed reference pct.** |
| --- | --- | --- | --- | --- | --- |
| PRJEB41443 | 89.72 | 3.13 | 1274 | 93.27 | 99.57 |
| PRJNA293971 | 93.12 | 1.54 | 3851 | 83.84 | 94.63 |
| PRJNA306884 | 95.09 | 2.15 | 1851 | 64.05 | 97.67 |
| PRJNA428736 | 95.54 | 2.96 | 7436 | 64.34 | 99.38 |
| PRJNA560950 | 95.22 | 1.39 | 1603 | 76.59 | 99.18 |
| PRJNA780023 | 83.63 | 6.79 | 2633 | 67.01 | 98.37 |
| PRJNA891951 | 70.86 | 6.33 | 10484 | 61.1 | 96.26 |
